# Gas signaling molecule: a new research focus in the treatment of erectile dysfunction

**DOI:** 10.4103/mgr.MEDGASRES-D-25-00333

**Published:** 2026-09-12

**Authors:** Xiaowei Dai, Zhenhuan Zhou

**Affiliations:** Reproductive Medical Center, Department of Obstetrics and Gynecology, the Second Norman Bethune Hospital of Jilin University, Changchun, Jilin Province, China

**Keywords:** CO, erectile dysfunction, gas signal molecule, H_2_S, molecular mechanism, nanocarrier, NO, refractory erectile dysfunction, combined therapy, targeted delivery

## Abstract

Gas signaling molecules, including nitric oxide, carbon monoxide, and hydrogen sulfide, have recently gained attention in erectile dysfunction treatment research because of their roles in regulating blood vessel function, reducing oxidative stress, and protecting nerves and other cells. This article reviews how nitric oxide, carbon monoxide, and hydrogen sulfide are involved in the development of erectile dysfunction and their potential as treatments. The review focuses on how these gases improve blood vessel function, relax smooth muscles, repair nerve function, and reduce tissue scarring. Current research shows that methods of gas delivery, especially those using nanocarriers or prodrugs, greatly increase how well these gases are absorbed and where they act in the body. Early animal studies and initial clinical trials support the effectiveness of these gasotransmitters in treating various causes of erectile dysfunction, including diabetes, aging, and nerve damage. Although challenges remain in controlling the speed of delivery, determining the appropriate dosage, and tailoring treatment to individual patients, there is reason for optimism. The development of responsive delivery systems, combinations of multiple gases, and additional therapies for difficult cases suggests that gas signaling molecules could transform erectile dysfunction treatment.

## Introduction

Erectile dysfunction (ED) is one of the most common male diseases. Due to the inability to achieve or maintain a penile erection of sufficient strength, it has a significant negative impact on the quality of life of patients. Epidemiological data show that about 50% of men over 40 years old have ED to varying degrees, and its prevalence increases with age.^1^

The treatment options for ED include psychological interventions, lifestyle modifications, oral medications, intracavernous drug injections, vacuum erection devices, shockwave therapy, and penile prosthesis implantation, among others. Currently, phosphodiesterase type 5 (PDE5) inhibitors (PDE5i), such as Sildenafil, Udinafil, and Tadalafil, are considered the first-line oral treatments for ED.^2^ The mechanism of action of PDE5i drugs is to enhance penile erectile hardness by increasing the level of cyclic guanosine monophosphate (cGMP). Due to the dependence of this class of drugs on the nitric oxide (NO) signaling pathway, the response rate to treatment in patients with chronic neurogenic ED is relatively low, and the clinical efficacy is still not satisfactory.^3^

The discovery of gas signaling molecules has provided a novel approach for treating ED. Gas signaling molecules are a class of endogenous gas mediators, including NO, carbon monoxide (CO), hydrogen sulfide (H_2_S), etc. These molecules play important physiological regulatory roles in the body and share the following common characteristics: small molecular weight, free penetration through biological membranes, short half-life, synthesis by specific enzymes, and concentration-dependent biological effects.^4^,^5^,^6^ In the male reproductive system, gas signaling molecules play a crucial role in the physiological process of penile erection by regulating vascular smooth muscle tone, endothelial function, and neural signal transmission.^4^,^5^,^6^ Therefore, in-depth exploration of the functions of the gas signaling molecule network in the pathophysiological mechanism of ED and the development of novel therapeutic strategies based on these molecules, such as regulating their synthesis, release, delivery or signaling pathways, have become a promising direction in the current ED research field, which is expected to provide innovative solutions to overcome the limitations of existing PDE5i treatments. The primary research purpose of this review is to systematically evaluate and synthesize the roles of gas signaling molecules, specifically NO, CO, and H_2_S, within the physiological and pathological frameworks of ED.

## Search Strategy

This review aims to comprehensively collect high-quality studies on the application of gaseous signaling molecules in the treatment of ED. A systematic search was conducted in the PubMed, Web of Science, Embase, Cochrane Library, and Google Scholar databases, covering the period from their inception to 2025. The search keywords fell into three categories: disease-related terms (erectile dysfunction, ED, penile erection, and refractory ED), molecule-related terms (gaseous signaling molecules, nitric oxide, carbon monoxide, hydrogen sulfide, molecular hydrogen), and technology- and mechanism-related terms (molecular mechanism, targeted delivery, nanocarriers, prodrugs, and PDE5i).

The inclusion criteria comprised: original studies investigating the role of gaseous molecules in the pathogenesis of ED; cutting-edge research on gaseous molecules and their donors, prodrugs, and delivery systems; animal models of ED induced by diabetes, aging, or nerve injury, as well as preliminary clinical evidence. Studies were excluded if they were duplicates, contained incomplete data, or were non-peer-reviewed or non-academic reports.

The initial search retrieved over 1200 relevant articles. After removing duplicates, news reports, and irrelevant reviews, approximately 350 studies were retained for further screening. Based on relevance, level of evidence, and innovation, this paper ultimately focuses on 163 core references, which are grouped into the following three categories: Basic mechanisms (emphasizing the NO-soluble guanylate cyclase (sGC)-cGMP pathway, the heme oxygenase (HO)/CO pathway, and the regulation of ion channels by H_2_S); delivery technologies (covering novel carriers such as light-controlled release systems, nanocapsules, and smart hydrogels); and clinical and translational research (including analyses of the limitations of PDE5i and the application prospects of novel gaseous donors in refractory ED).

## Physiological Mechanism of Penile Erection

Penile erection is a highly coordinated physiological process involving the precise collaboration of the nervous, vascular, and endocrine systems and the corpus cavernosum tissue. The core of this process lies in the relaxation of the corpus cavernosum smooth muscle and significant changes in hemodynamics. This process can be regulated through multi-level mechanisms, including signal transmission in the central and peripheral nervous systems, the release of vasoactive substances, the activation of intracellular signaling pathways, and the adaptive remodeling of the corpus cavernosum structure.^7^

### Neural regulatory network

The neural regulation of penile erection can be divided into two systems: central regulation and peripheral regulation. The central regulation system is mainly located in the limbic system of the brain, the hypothalamus and the brainstem, responsible for integrating sexual stimulation signals from sources such as vision, hearing, touch and psychological activities, and transmitting impulses to the spinal erection center (T11–L2 and S2–S4) through descending conduction pathways, such as the spinothalamic tract and reticulospinal tract. Among them, the sacral parasympathetic nucleus is the key center for initiating erection, and its efferent nerve impulses are conducted to the corpus cavernosum of the penis through the pelvic nerve.^8^,^9^

The peripheral nerves mainly include parasympathetic nerves, sympathetic nerves and somatic nerves. The parasympathetic nerves (mainly pelvic nerves) play a leading role in the process of sexual stimulation. Their terminals release acetylcholine, which promotes the synthesis of NO by activating M3 receptors on vascular endothelial cells, and can directly act on the smooth muscle of the penile corpus cavernosum to induce relaxation.^10^ The sympathetic nerve (hypogastric nerve) is dominant during the detumescence period, releasing norepinephrine, which mediates smooth muscle contraction through α1 receptors and reduces blood perfusion. The somatic nerve (pudendal nerve) is responsible for the sensory input of the penis and the contraction of the bulbocavernosus muscle, participating in the maintenance of erection and the ejaculation process.^11^,^12^

Recent studies have shown that non-adrenergic non-cholinergic nerves play a significant role in the regulation of penile erectile function. The nerve terminals of non-adrenergic non-cholinergic can release various neuropeptides such as vasoactive intestinal peptide and calcitonin gene-related peptide, which promote smooth muscle relaxation by activating the cyclic adenosine monophosphate signaling pathway and enhance the erectile effect in synergy with NO. In addition, purine substances, such as adenosine and adenosine triphosphate (ATP), as neurotransmitters of the non-adrenergic non-cholinergic system can regulate potassium ion channels and participate in the regulation of smooth muscle tension in the corpus cavernosum of the penis^13^,^14^ (**Figure 1**).

**Figure 1:**
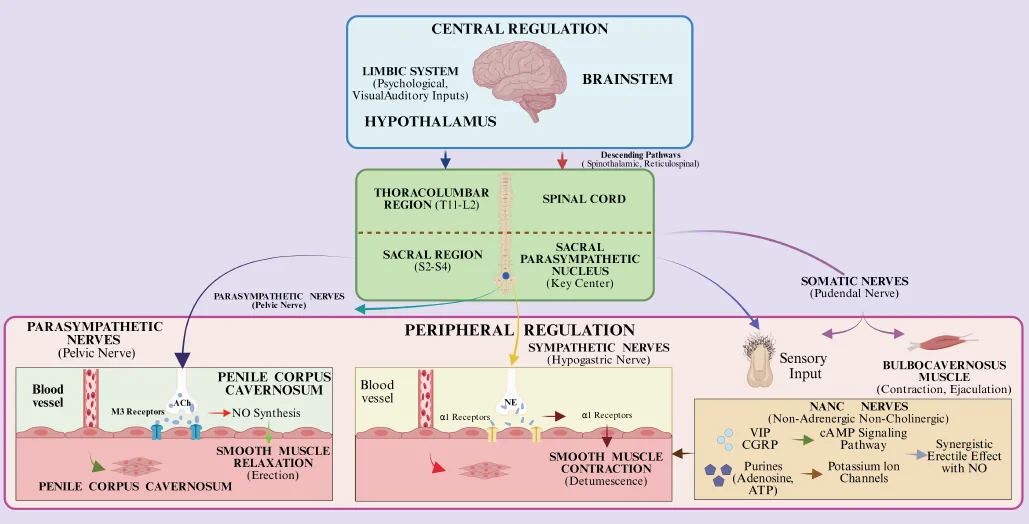
Neural regulatory network of penile erection. The central system (limbic system, hypothalamus, and brainstem) is responsible for integrating multisource sensory signals and transmitting them via descending pathways to the spinal erection centers (T11–L2 and S2–S4), with the sacral parasympathetic nuclei initiating erection through the pelvic nerves. In peripheral regulation, parasympathetic nerves release ACh to activate M3 receptors and promote NO synthesis for smooth muscle relaxation, while sympathetic nerves maintain detumescence through NE and α1 receptors, and somatic nerves are responsible for sensory input and muscle contraction. Additionally, the NANC system enhances erectile function via VIP, CGRP, and purine substances, working synergistically with NO through the cAMP pathway and potassium channels. Created with BioRender.com. Ach: Acetylcholine; ATP: adenosine triphosphate; cAMP: cyclic adenosine monophosphate; CGRP: calcitonin gene-related peptide; NANC: non-adrenergic, non-cholinergic; NE: norepinephrine; NO: nitric oxide; VIP: vasoactive intestinal peptide.

### Synergistic effects of vascular and corpus cavernosum tissues

The corpus cavernosum is composed of interconnected sinusoids and is surrounded by a dense and tough tunica albuginea, which mainly consists of collagen and elastic fibers. During erection, the dilation of the cavernous arteries (branches of the deep penile artery) and their helical branches is a key step in achieving penile blood perfusion.^15^ When sexual stimulation is triggered, the smooth muscle of the helical arteries relaxes, and the lumen diameter can expand by 3 to 4 times. The blood flow rate sharply increases from 2 to 5 mL/min at the resting state to 100 to 200 mL/min, thereby promoting the rapid filling and expansion of the sinusoids.^16^

As the pressure within the sinusoids increases, the trabeculae of the corpus cavernosum are stretched, which in turn compresses the venous plexus beneath the tunica albuginea, resulting in obstruction of venous return (venous occlusion mechanism). This mechanism is the core link in maintaining an erect penile state.^17^,^18^ The biomechanical properties of the tunica albuginea play a crucial role in this process: the longitudinally arranged collagen fibers in the inner layer and the circumferentially arranged fibers in the outer layer can produce a “constriction effect” when the pressure in the corpus cavernosum increases, further restricting venous return and raising the intracavernous pressure to a level close to the systolic blood pressure (about 100 mmHg), thereby achieving a firm and erect penis.^17^,^18^ Research indicates that the phenotypic transformation of smooth muscle cells in the corpus cavernosum may be involved in the regulation of erectile function. Under normal conditions, smooth muscle cells mainly exhibit a contractile phenotype; however, during an erection, some cells can temporarily transform into a synthetic phenotype, secreting extracellular matrix components, such as elastin, to accommodate the expansion of the tissue.^19^

### Nitric oxide signaling pathway in the regulation of erectile function

NO as an endogenous vasodilator, is a core molecule in the regulation of erectile function. Its synthesis is catalyzed by NO synthase (NOS) from L-arginine. Neuronal NOS (nNOS) is mainly distributed in parasympathetic nerve endings, while endothelial NOS (eNOS) is expressed in vascular endothelial cells. Under sexual stimulation, nNOS is activated by calmodulin and releases NO, while eNOS is activated by the shear stress caused by increased blood flow, jointly promoting the synthesis of NO.^20^

NO diffuses into smooth muscle cells and binds to the heme group of sGC, inducing a conformational change and activation of sGC, which catalyzes the conversion of guanosine triphosphate to cGMP. As a second messenger, cGMP triggers a series of cascading effects by activating protein kinase G (PKG). Phosphorylation of voltage-gated calcium channels leads to their closure, reducing Ca^2+^ influx; enhanced activity of the sarcoplasmic reticulum calcium pump promotes Ca^2+^ reuptake; inhibition of myosin light chain kinase activity reduces the phosphorylation level of myosin light chain, ultimately causing smooth muscle relaxation^21^,^22^,^23^ (**Figure 2**).

**Figure 2:**
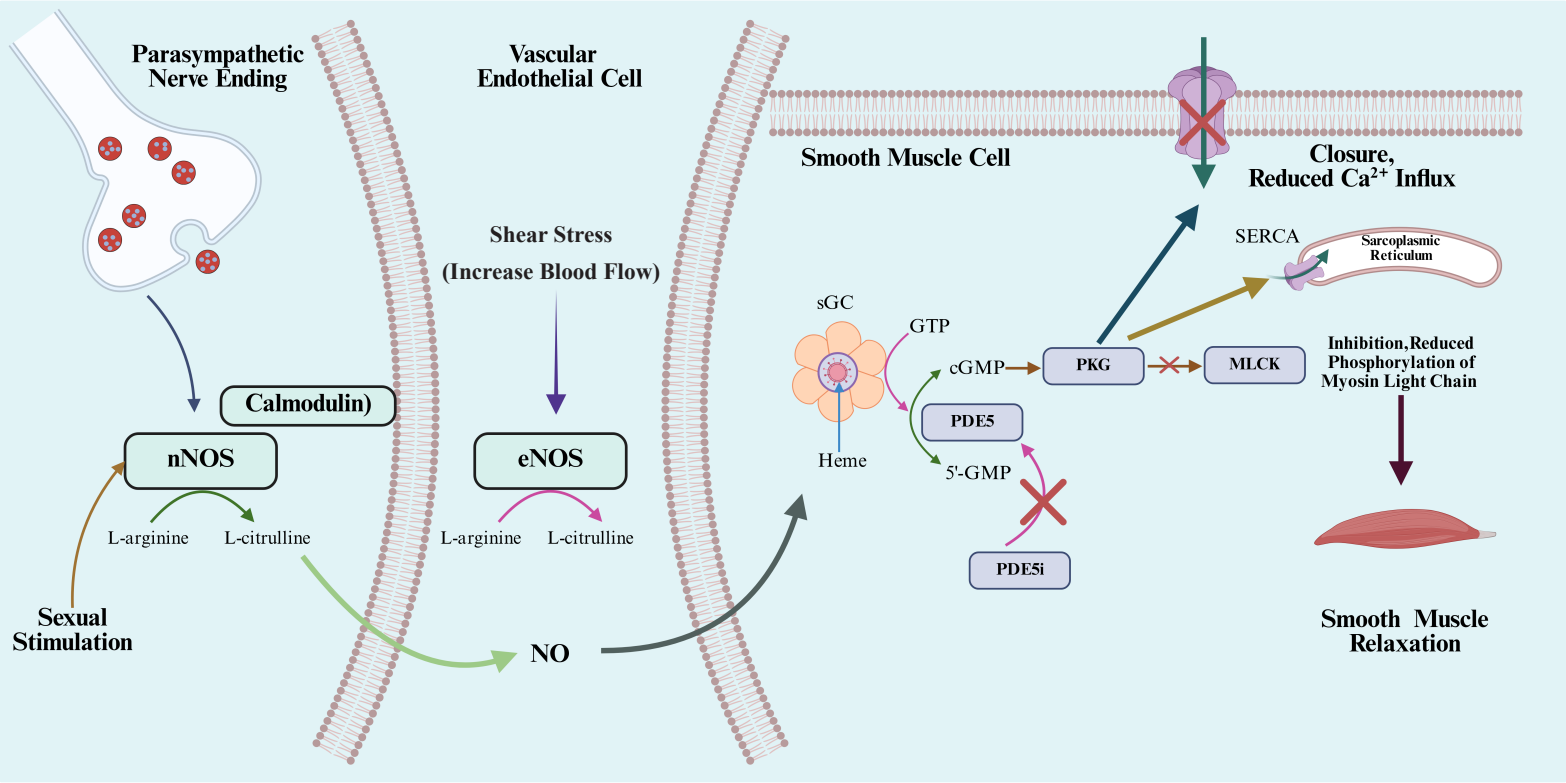
The sexual stimulation triggered NO-sGC-cGMP pathway and its downstream regulatory processes. In parasympathetic nerve terminals and vascular endothelial cells, nNOS and eNOS convert L-arginine into NO. NO diffuses into smooth muscle cells and activates sGC, which catalyzes the conversion of GTP to cGMP. Subsequently, cGMP activates PKG, which lowers intracellular Ca^2+^ concentration through the following three mechanisms: (1) inhibiting VGCC to reduce calcium influx; (2) enhancing SERCA activity to promote calcium reuptake; and (3) inhibiting MLCK activity. These pathways ultimately lead to smooth muscle relaxation and penile erection. In addition, PDE5 is responsible for degrading cGMP into 5′-GMP, and PDE5i maintain high cGMP levels by blocking this degradation process, thereby improving erectile function. Created with BioRender.com. 5′-GMP: Guanosine 5′-monophosphate; cGMP: cyclic guanosine monophosphate; eNOS: endothelial nitric oxide synthase; GTP: guanosine triphosphate; MLCK: myosin light-chain kinase; nNOS: neuronal nitric oxide synthase; NO: nitric oxide; PDE5: phosphodiesterase type 5; PDE5i: phosphodiesterase type 5 inhibitors; SERCA: sarco/endoplasmic reticulum Ca^2+^-ATPase; sGC: soluble guanylyl cyclase; VGCC: voltage-gated calcium channel.

### Regulatory role of endocrine and metabolic factors

Androgens, mainly testosterone, are fundamental sex hormones that maintain erectile function. Their effects are multi-faceted: they promote the expression and activity of nNOS and eNOS, thereby enhancing NO synthesis; they upregulate the expression of androgen receptors in penile smooth muscle cells, maintaining the normal phenotype and functional stability of the cells; and they regulate the sex drive-related neural circuits in the central nervous system, enhancing the integration and transmission of sexual stimulation signals.^24^,^25^ When testosterone levels fall below 300 ng/dL, approximately 40% of men will experience ED. Testosterone supplementation therapy can improve erectile function in some patients, but it is usually ineffective for ED patients with normal androgen levels.^26^,^27^

Estrogen plays a dual role in the regulation of erectile function: at low concentrations, it can promote the expression of eNOS through estrogen receptor α, enhancing the smooth muscle relaxation mediated by NO; while at high concentrations, it may inhibit the smooth muscle relaxation response through estrogen receptor β, exerting an opposite effect.^28^,^29^ Clinical studies have shown that an elevated estrogen/testosterone ratio in middle-aged and elderly men is significantly associated with an increased risk of ED, suggesting the importance of maintaining hormonal balance in the regulation of erectile function.^25^,^30^

Metabolic factors such as blood glucose and lipid levels can indirectly affect the erectile process by damaging vascular endothelial function. Hyperglycemia promotes the accumulation of advanced glycation end products, inhibits eNOS activity, and induces oxidative stress responses, such as increased superoxide anion production, accelerating the degradation and inactivation of NO. Hyperlipidemia, on the other hand, impairs the barrier function of endothelial cells and reduces the bioavailability of NO.^31^ These mechanisms collectively explain the higher incidence of ED in patients with diabetes and atherosclerosis.

Penile erection is a complex physiological process involving the highly coordinated interaction of nerves, blood vessels and molecular signaling networks (**Figure 3**).

**Figure 3:**
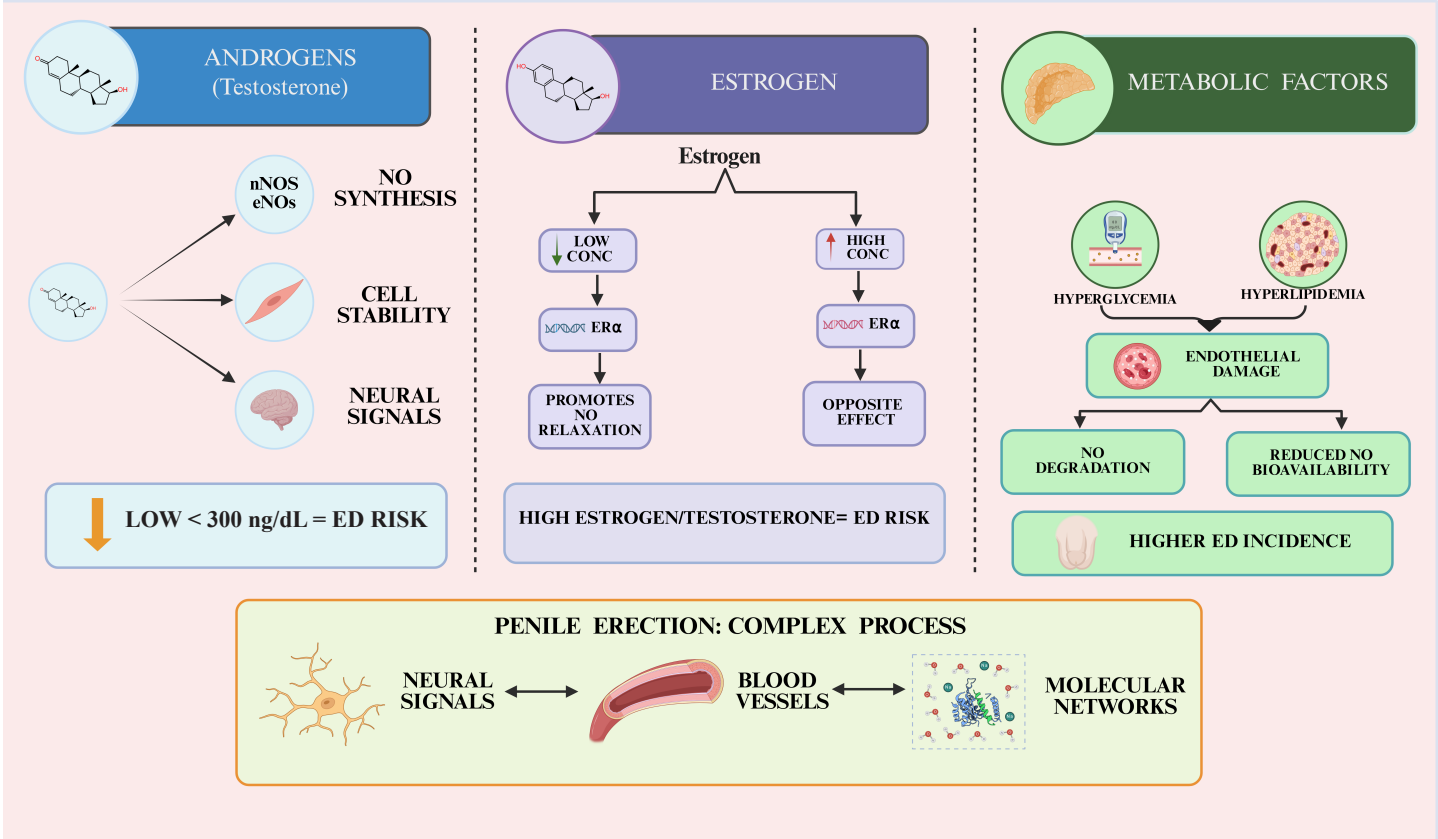
The molecular mechanisms on endocrine and metabolic factors regulate erectile function. Androgens play a key positive role by enhancing nNOS/eNOS expression, maintaining smooth muscle phenotype stability, and regulating central neural circuits; estrogens exhibit concentration-dependent bidirectional regulatory characteristics, with low concentrations promoting erection synergistically through ERα, while high concentrations may exert inhibitory effects via ERβ, highlighting the importance of hormonal balance; metabolic factors reduce NO bioavailability by inducing oxidative stress, accumulating AGEs, and damaging the endothelial barrier, elucidating the molecular mechanisms by which diabetes and atherosclerosis lead to ED. Created with BioRender.com. AGE: Advanced glycation end-products; CONC: concentration; ED: erectile dysfunction; eNOS: endothelial nitric oxide synthase; ER: estrogen receptor; nNOS: neuronal nitric oxide synthase.

## Pathogenesis of Erectile Dysfunction

### Vascular factors

Vascular factors are the leading cause of organic ED, accounting for 50–70%. The core pathological mechanism lies in “insufficient blood supply” or “abnormal venous return” to the corpus cavernosum of the penis. According to the pathological characteristics, it can be classified into three types: arterial ED, venous ED and vascular endothelial dysfunction.^24^ The mechanism of arterial ED is based on the dependence of penile erection on adequate blood flow perfusion of the cavernous arteries (mainly the branches of the deep penile artery) and the helical arteries. When diseases such as atherosclerosis, hypertension, diabetes, or hyperlipidemia cause vascular stenosis or occlusion, the arterial blood flow cannot meet the sharp increase required for erection. If the vascular dilation capacity is impaired due to arterial disease, it will directly cause “insufficient blood supply” and make it difficult to effectively fill the sinusoids. For example, in diabetic patients, long-term hyperglycemia can cause vascular endothelial damage, which can reduce the elasticity of the cavernous arteries and decrease blood flow perfusion by more than 30%.^32^ The key pathological mechanism of venous ED lies in the dysfunction of the venous occlusion mechanism. The maintenance of penile erection depends on the compression of the venous plexus under the tunica albuginea after the sinusoids are filled, thereby blocking blood reflux. When the structure of the tunica albuginea is abnormal, such as collagen fiber hyperplasia and elastic fiber reduction, the corpus cavernosum trabeculae are damaged, such as trabecular fibrosis, or there is a congenital venous fistula, the venous reflux cannot be effectively inhibited, resulting in “uncontrolled reflux.” For example, the tear of the tunica albuginea caused by penile trauma can destroy the venous occlusion structure. Even if the arterial blood supply is normal, it is difficult to maintain sufficient intracavernous pressure, usually above 100 mmHg to achieve a rigid erection, and ultimately manifests as inadequate erection or easy to become flaccid.^33^ Vascular endothelial dysfunction is one of the important mechanisms of ED. As the “signal center” for regulating blood flow, vascular endothelium can mediate vascular dilation responses by releasing NO. When the endothelium is damaged by factors such as hyperglycemia, oxidative stress or inflammation, the activity of eNOS decreases, leading to reduced NO synthesis and decreased bioavailability. This, in turn, weakens the ability of vascular smooth muscle to dilate, exacerbating arterial insufficiency. The above changes can form a vicious cycle of “endothelial injury, reduced NO, restricted vascular dilation, and further deterioration of blood perfusion,” continuously damaging erectile function.^34^

### Neurological factors

Penile erection depends on the coordinated transmission of “sexual stimulation signals” by the central and peripheral nervous systems. Any nerve damage or functional disorder in any link can lead to the interruption of signal transmission, which may involve abnormalities in the central nervous system, peripheral nervous system and neurotransmitter system.^35^,^36^ Central nervous system disorders can significantly affect erectile function. The limbic system, hypothalamus and brainstem, as signal integration centers, receive multimodal stimulation signals from vision, hearing and psychology, and transmit them to the spinal cord erectile center (T11–L2 and S2–S4). When there are diseases such as brain tumors, cerebrovascular accidents, such as cerebral infarction, multiple sclerosis or Parkinson’s disease, the ability of central signal integration is impaired, leading to sexual excitation conduction disorders. For example, in patients with Parkinson’s disease, due to the degeneration and damage of dopaminergic neurons in the brainstem, the down-conduction of sexual stimulation signals is blocked, and the incidence of ED is as high as 60% to 80%.^37^,^38^

### Psychological factors

Psychological factors account for 20% to 30% of ED cases. The core mechanism is that negative psychological states inhibit and interfere with the neural signal transmission related to erection through the central nervous system, rather than being caused by abnormal organ structures. Common triggers include emotional disorders, cognitive biases, and interpersonal relationship issues.^39^ Anxiety, depression and tension are the main causes of psychogenic ED. Anxiety, such as fear of erectile failure, can activate the “inhibitory center” in the cerebral cortex, thereby inhibiting the signal output of the spinal erectile center, resulting in a decrease in parasympathetic nerve excitability and a reduction in NO synthesis; depression affects the effective integration of sexual stimulation signals in the central nervous system by reducing dopamine levels (dopamine is a key sexual drive neurotransmitter), and the clinical manifestations are lack of erectile desire or inability to maintain an erection.^40^,^41^ Negative perceptions of sexual ability, such as the belief that aging inevitably leads to ED, and previous negative sexual experiences, such as self-denial after erectile failure, can form a continuous psychological suggestion, causing individuals to overly focus on the evaluative judgment of whether it is normal during sexual activities rather than the sexual stimulation itself, thereby interfering with the normal sexual response process and further intensifying the inhibitory effect of the central nervous system.^42^,^43^ Tense relationships between partners, such as poor communication and emotional conflicts, and an unsatisfactory sexual living environment, such as a lack of privacy, can reduce the “pleasure” of sexual stimulation, making it difficult for the brain to effectively receive and integrate positive sexual-related signals, thereby indirectly inhibiting the erectile response.^44^

### Other factors

Aging, smoking, excessive drinking, side effects of medication, and chronic diseases, such as chronic renal insufficiency and chronic liver insufficiency, are all closely related to the occurrence of ED.^45^ Aging can lead to the decline of the function of smooth muscle cells and endothelial cells in the corpus cavernosum, and the reduction of the synthesis and release of NO, thereby increasing the risk of ED. Smoking and excessive drinking can damage the function of vascular endothelium and affect the blood supply to the corpus cavernosum, which in turn can cause ED. Some drugs, such as antihypertensive drugs, antidepressants and antipsychotic drugs, can cause ED by interfering with the synthesis, release or receptor action of neurotransmitters.^46^,^47^

The pathogenesis of ED is a multidimensional pathophysiological process driven by an imbalance between vascular function, neural conduction, psychological states, and overall systemic health. At its core, the physical mechanism involves vascular failure, characterized by “insufficient arterial inflow” or “venous leakage,” which is frequently triggered by a reduction in NO synthesis due to endothelial damage. This is often compounded by neurological impairments that interrupt the transmission of sexual stimuli and psychological factors (such as anxiety or depression) that exert inhibitory control via the central nervous system. Furthermore, ageing, chronic diseases, and poor lifestyle habits (like smoking and excessive drinking) form a detrimental network that damages the cavernous smooth muscle and circulatory system, ultimately preventing the achievement or maintenance of sufficient penile rigidity^48^,^49^,^50^ (**Figure 4**).

**Figure 4:**
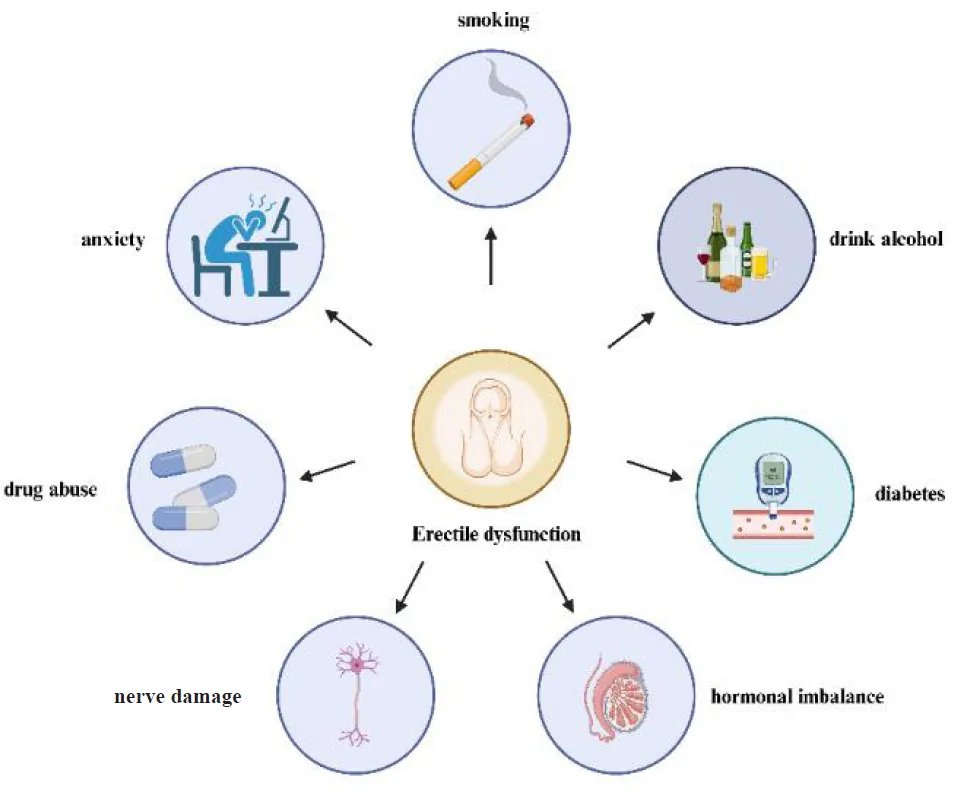
Causes of erectile dysfunction Penile erection is a complex physiological phenomenon governed by the seamless integration of neurogenic and vasculogenic signaling networks. Simultaneously, endocrine factors, particularly androgens, and metabolic determinants modulate the functional integrity of this regulatory system through sustained homeostatic maintenance. This multifaceted synergy ensures that the erectile tissue can respond dynamically to acute stimuli while preserving its structural and functional capacity over the long term. Created with BioRender.com.

## Mechanism of Gas Signaling Molecules in Penile Erection

### Nitric oxide

NO is a lipid-soluble gas molecule with high biological activity and diffusion capacity, which can rapidly pass through cell membranes and play a signaling role locally. It is generated by the oxidation of the guanidine nitrogen atom of L-arginine catalyzed by NOS, while producing the by-product citrulline. This reaction requires the participation of multiple cofactors, including nicotamide adenine dinucleotide phosphate, flavin adenine dinucleotide, flavin mononucleotide, tetrahydrobiopterin, and oxygen, demonstrating its complex enzymatic mechanism.^51^ There are three subtypes of NOS, namely nNOS, iNOS and eNOS. Although these three subtypes catalyze the same biochemical reaction, that is, converting L-arginine into NO and citrulline, they have significant differences in gene structure, tissue distribution, regulatory mechanisms and physiological functions. nNOS is mainly located in the central and peripheral nervous systems, especially highly expressed in the brain, spinal cord and nerve endings of the penile corpus cavernosum.^52^ Its activity is regulated by calcium ions and calmodulin, and it is usually rapidly activated when a nerve impulse arrives, releasing NO as a neurotransmitter or paracrine factor to participate in rapid physiological processes such as synaptic plasticity, learning and memory, and the erectile reflex. eNOS is mainly expressed in vascular endothelial cells and is also activated by calcium ions. Under physiological conditions, it continuously produces low levels of NO, playing a protective role in maintaining vascular tone, inhibiting platelet aggregation and preventing leukocyte adhesion. During penile erection, NO derived from eNOS is a key initiating signal for the relaxation of corpus cavernosum smooth muscle, and is therefore closely related to erectile function.^53^ In contrast, iNOS is not continuously expressed under normal physiological conditions, but is induced by various cells such as macrophages, neutrophils and smooth muscle cells after being stimulated by inflammation, such as lipopolysaccharide, tumor necrosis factor or interferon-γ and other cytokines. Once synthesized, iNOS can produce large amounts of NO for a long time without the dependence on calcium ions, participating in host defense and immune regulation. However, excessive or continuous production of NO may also cause tissue damage and chronic inflammation, and is associated with various pathological conditions.^51^,^54^ In the corpus cavernosum of the penis, nNOS is mainly distributed in nerve endings, eNOS is mainly located in endothelial cells, while iNOS has a low expression level under normal physiological conditions and can only be induced to express under pathological conditions such as inflammation. The half-life of NO is extremely short, and it is usually rapidly oxidized to nitrate and nitrite within a few seconds and becomes inactive.^55^ During penile erection, sexual stimulation can activate the parasympathetic nerve endings, non-adrenergic non-cholinergic nerve endings and vascular endothelial cells in the corpus cavernosum of the penis, promoting these cells to synthesize and release NO under the catalytic action of NOS.^56^ After NO diffuses into the smooth muscle cells of the penile corpus cavernosum, it rapidly crosses the cell membrane due to its lipid solubility and specifically binds to sGC in the cytoplasm. This binding causes a conformational change in sGC and activates it, enabling its catalytic function to convert guanosine triphosphate into cGMP. As an important second messenger, the increase in intracellular cGMP concentration further activates PKG, initiating a series of downstream signaling cascades.^57^ The activation of PKG can reduce intracellular free calcium ion (Ca^2+^) concentration through multiple pathways: on the one hand, it promotes the uptake of calcium ions into the sarcoplasmic reticulum for storage; on the other hand, it activates potassium channels to cause cell membrane hyperpolarization, inhibiting the opening of voltage-dependent calcium channels and reducing calcium ion efflux. Additionally, PKG can phosphorylate myosin light chain phosphatase, inhibiting the activity of myosin light chain kinase, disrupting the phosphorylation balance required for smooth muscle contraction, and ultimately promoting smooth muscle relaxation.^58^ With the widespread relaxation of the corpus cavernosum smooth muscle, the sinusoids open, the resistance of small arteries significantly decreases, and a large amount of arterial blood rapidly flows into the corpus cavernosum cavity, causing it to fill and expand, and compressing the venous return system under the tunica albuginea, forming a “venous occlusion mechanism,” thereby maintaining a sustained and sufficient erectile state. It is worth noting that this NO-sGC-cGMP signaling pathway is not only the core regulatory mechanism for penile erection under physiological conditions but also the target of currently widely used PDE5i in clinical practice. These drugs inhibit the degradation of cGMP, enhance and prolong the NO signaling effect, and improve the hemodynamic response in patients with ED.^59^,^60^

Vascular endothelial dysfunction is one of the important pathological mechanisms leading to ED, and the reduction in the synthesis and release of NO is the core manifestation of vascular endothelial dysfunction.^61^ Research indicates that in chronic disease models closely related to ED, such as diabetes, hypertension, and atherosclerosis, the activity of NOS in the corpus cavernosum tissue is significantly reduced, leading to a decrease in NO production and a subsequent decline in cGMP levels, ultimately resulting in impaired erectile function. The core mechanism of these changes lies in the progressive damage to vascular endothelium and nerve function.^62^ For instance, in diabetes, long-term hyperglycemia can induce oxidative stress, increase the production of reactive oxygen species (ROS), and accelerate the degradation of NO; meanwhile, the accumulation of advanced glycation end products can inhibit the expression and activity of eNOS and disrupt its normal phosphorylation activation process.^31^ Additionally, diabetes is often accompanied by autonomic neuropathy, which affects the function of nNOS-positive nerve fibers and weakens the release of neurogenic NO, further hindering the transmission of erectile initiation signals. In patients with hypertension, continuous hemodynamic pressure can cause thickening of the penile arteriole walls and disordered endothelial cell structure, leading to eNOS uncoupling, which not only reduces NO synthesis but also promotes the generation of superoxide anions, creating a vicious cycle.^63^ Atherosclerosis, on the other hand, directly limits the blood perfusion capacity of the penile arteries through plaque formation and vascular lumen narrowing, while the NO bioavailability in the diseased vascular endothelium significantly decreases, affecting the smooth muscle relaxation effect.^64^ Notably, these pathological states often act together on the key links of the NO-sGC-cGMP signaling pathway: inhibited NOS activity leads to insufficient NO synthesis; excessive ROS rapidly clear the generated NO; and relatively enhanced PDE5 activity accelerates the hydrolysis of cGMP. The combined effect of these three factors results in cGMP concentrations failing to reach the effective threshold, making it difficult to maintain sufficient smooth muscle relaxation and cavernous engorgement, thereby triggering or exacerbating ED.^65^ Exogenous administration of NO donors or increasing the intake of its precursor L-arginine can effectively enhance the NO level in the corpus cavernosum, thereby improving erectile function. NO donor drugs such as sodium nitroprusside and nitroglycerin can release NO under non-enzymatic conditions in the body, directly activating sGC and promoting cGMP production, mimicking the physiological effects of NO from neural and endothelial sources. L-arginine, as the substrate for the NOS catalytic reaction, can increase NO synthesis efficiency to a certain extent when supplemented, especially showing potential as an adjuvant treatment in patients with mild endothelial dysfunction. However, a clinical study has found that in pathological conditions such as severe diabetes or atherosclerosis, which significantly reduce NOS activity, the effect of simply supplementing L-arginine is limited, suggesting that substrate sufficiency cannot completely reverse enzyme dysfunction.^66^ Additionally, PDE5i, by selectively inhibiting the hydrolysis of cGMP, extends its half-life in penile smooth muscle cells, thereby amplifying and maintaining the NO-induced relaxation signal. These drugs only work under sexual stimulation and rely on endogenous NO release, thus essentially enhancing the function of the complete NO-sGC-cGMP pathway. Currently, PDE5i, due to their oral convenience and generally good safety profile, have become the first-line treatment for ED and are widely used worldwide. However, some ED patients show poor or no response to PDE5i treatment, with an overall response rate of 60–70%, especially common in those with diabetes, hypertension, cardiovascular disease, or a history of pelvic surgery.^61^ Studies have shown that this treatment resistance is mainly related to severe damage to key links in the NO signaling pathway: for example, down-regulation of eNOS expression or “uncoupling” of the enzyme, leading to reduced NO production; degeneration of nNOS nerve fibers affecting neurogenic NO release; and increased oxidative stress rapidly clearing NO with superoxide anions (O_2_^−^) to form peroxynitrite (ONOO^−^), further damaging tissue structure. Even if PDE5 is effectively inhibited, due to insufficient upstream cGMP production, downstream effects are still difficult to achieve, resulting in a “target loss” state.^63^ For such refractory ED, the clinical field is exploring various alternative or combined strategies, including using sGC agonists, such as Riociguat, to bypass NO and directly activate sGC; intracavernous injection of vasoactive drugs, such as Alprostadil; and applying low-intensity extracorporeal shock wave therapy to promote angiogenesis and endothelial function repair. These methods aim to restore or rebuild the functional basis of the NO signaling pathway, providing new intervention approaches for patients unresponsive to traditional treatments. In summary, although PDE5i play a central role in ED treatment, their efficacy is highly dependent on the integrity of the NO pathway. Future individualized treatment strategies should combine etiological assessment and focus on functional reconstruction at different nodes of the pathway^67^,^68^ (**Figure 5**).

**Figure 5:**
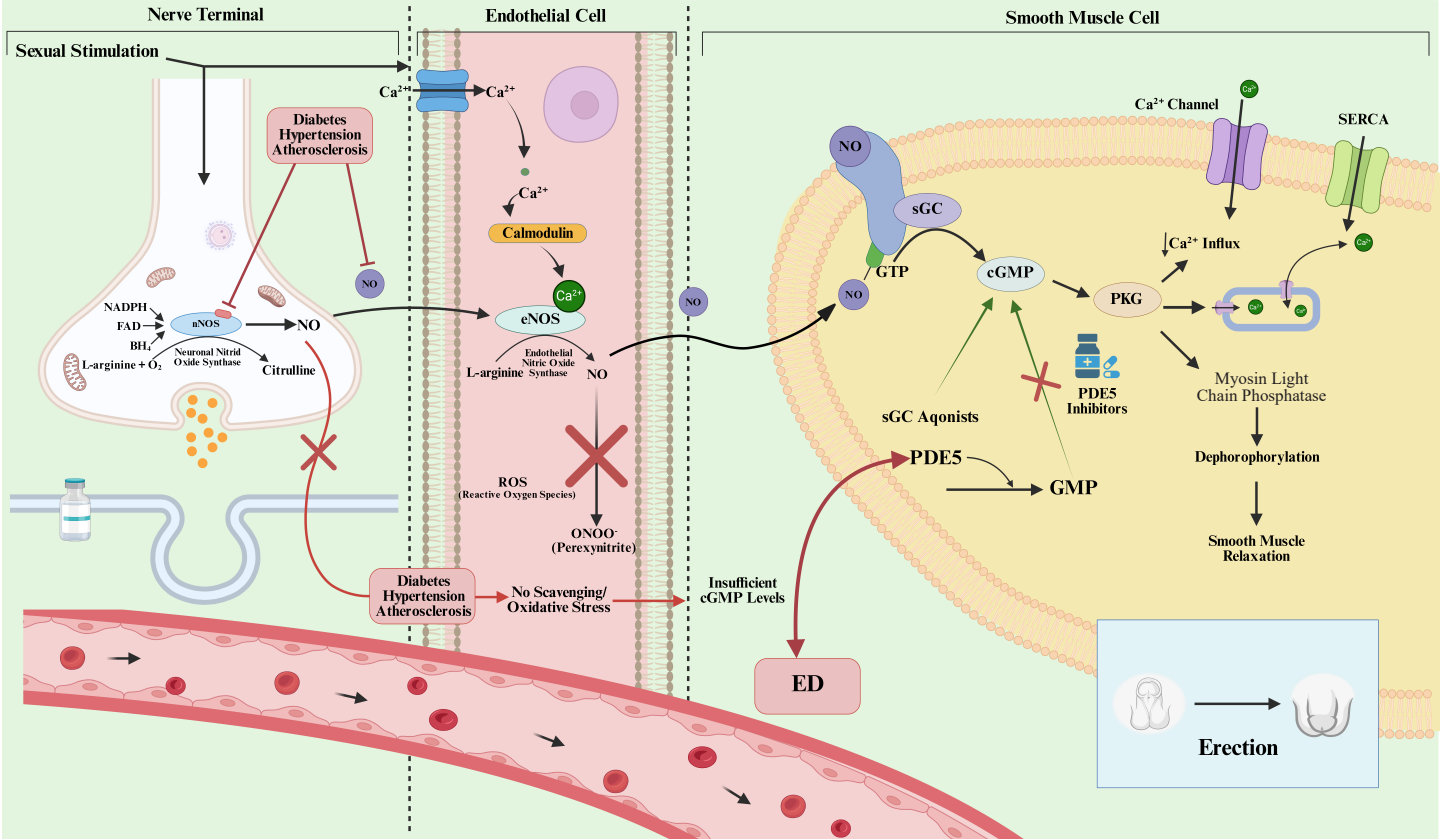
The core mechanism of NO in the corpus cavernosum of the penis. Sexual stimulation prompts nNOS and eNOS to use L-arginine to synthesize NO. NO diffuses into the smooth muscle cells of the corpus cavernosum, activates sGC, which catalyzes the conversion of GTP to the second messenger cGMP. cGMP activates PKG, leading to a decrease in intracellular calcium concentration, causing smooth muscle relaxation, vasodilation, and ultimately erection. Pathological factors such as diabetes, hypertension, and oxidative stress can reduce NO bioavailability, leading to insufficient cGMP and resulting in ED. PDE5 inhibitors maintain cGMP levels by blocking its degradation by PDE5, while NO donors and sGC agonists directly enhance this signaling pathway. Created with BioRender.com. BH4: Tetrahydrobiopterin; cGMP: cyclic guanosine monophosphate; ED: erectile dysfunction; eNOS: endothelial nitric oxide synthase; FAD: flavin adenine dinucleotide; GTP: guanosine triphosphate; NADPH: nicotinamide adenine dinucleotide phosphate; nNOS: neuronal nitric oxide synthase; NO: nitric oxide; O_2_: oxygen; ONOO^−^: peroxynitrite; PDE5: phosphodiesterase type 5; PKG: cGMP-dependent protein kinase; SERCA: sarco/endoplasmic reticulum Ca^2+^-ATPase; sGC: soluble guanylyl cyclase.

### Carbon monoxide

CO is mainly produced by the degradation of heme catalyzed by HO. This reaction not only generates CO but also releases an equal molar amount of biliverdin and free iron ions. Biliverdin can be rapidly reduced to bilirubin by biliverdin reductase, which has significant antioxidant activity, while free iron can induce the expression of ferritin and participate in the regulation of intracellular iron homeostasis. Therefore, the heme decomposition mediated by HO is not only a key metabolic pathway for the body to clear hemoglobin in senescent red blood cells, but also an important source of various bioactive molecules, playing multiple physiological functions in cell protection, anti-inflammatory responses and signal transduction processes.^69^

There are three isoenzymes of HO: HO*-*1, HO-2 and HO-3. Among them, HO*-*1 is a highly inducible stress response protein belonging to the heat shock protein family. Under various pathological or physiological stimuli such as oxidative stress, inflammatory factor stimulation, ultraviolet radiation, heavy metal exposure, hypoxia and hyperglycemia, its expression level can be significantly upregulated through the activation of nuclear factor erythroid 2-related factor 2/antioxidant response element, AP-1 and nuclear factor kappa-B signaling pathways. HO-1 is widely distributed in tissues such as the liver, spleen, macrophages and vascular endothelial cells. Its inducible expression is regarded as an important endogenous defense mechanism, which can reduce tissue damage, inhibit inflammatory responses and promote cell survival by generating CO and other metabolic products. HO-2 is a constitutive enzyme that is continuously and stably expressed under physiological conditions, and its activity is not significantly affected by external stress factors. It is mainly located in the central nervous system, peripheral nervous system and testicular tissue, with higher content in the hippocampus, hypothalamus and autonomic ganglia. The activity of HO-2 is regulated by calmodulin and may be involved in physiological processes such as neurotransmission, synaptic plasticity regulation and cerebral blood flow control. Recent studies have shown that CO derived from HO-2 can promote the relaxation of penile corpus cavernosum smooth muscle by activating the sGC-cGMP signaling pathway, playing a certain role in regulating erectile function, suggesting its potential physiological significance in the male reproductive system. HO-3 has a high amino acid sequence homology with HO-2 and was initially considered to be a pseudogene product of HO-2. Its coding region contains multiple non-functional mutations, resulting in low translation efficiency. Although some studies have detected the mRNA expression of HO-3 in certain tissues, its protein expression level is extremely low and it lacks obvious catalytic activity. Currently, it is believed that HO-3 may not have typical heme degradation function, and its biological role is more likely to be a regulatory molecule involved in gene expression regulation or protein-protein interactions. The specific mechanism remains to be further clarified.^70^,^71^

CO plays a significant role in the process of penile erection and is one of the gas signal molecules that have attracted much attention in the field of sexual physiology in recent years. Similar to the classic neurotransmitter NO, CO is regarded as an endogenous gas transmitter that can diffuse in local tissues and participate in intercellular signal transmission. Studies have shown that CO can activate sGC in penile corpus cavernosum smooth muscle cells, promoting the conversion of guanosine triphosphate to cGMP. As a second messenger, cGMP triggers a series of downstream effects within the cell, including reducing the concentration of cytoplasmic calcium ions, ultimately leading to smooth muscle relaxation and increased blood flow perfusion in the penile arteries, creating the necessary physiological conditions for penile erection.^72^ In addition to exerting its effects through the sGC-cGMP pathway, CO can also cause hyperpolarization of the smooth muscle cell membrane by regulating the activity of various potassium channels, such as ATP-sensitive potassium channels and calcium-activated potassium channels, thereby altering the cell membrane potential, inhibiting the opening of voltage-dependent calcium channels, reducing calcium ion influx, and further promoting the relaxation of penile corpus cavernosum smooth muscle. This dual mechanism of action enhances the biological significance of CO in regulating penile vascular tone and erectile function. It is worth noting that CO does not exist directly in the body but is generated by the degradation of heme catalyzed by HO. In penile corpus cavernosum tissue, both of the two main isoenzymes of HO, HO-1 and HO-2, are expressed, but HO-2 is constitutively expressed and mainly located in neural tissue, thus being considered to play a dominant role in the physiological regulation of erection. Multiple experiments have observed that HO-2 is highly expressed in the nerve endings of the penile corpus cavernosum, and its activity is closely related to the state of neural excitation. When electrical stimulation is given to simulate nerve impulses, the expression level of HO-2 can be significantly upregulated, thereby promoting the synthesis and release of CO and enhancing the erectile response. Animal model studies have also shown that inhibiting HO activity leads to weakened erectile function, while enhancing the HO/CO pathway can improve ED. Moreover, there are interactions between the HO/CO system and other known erectile regulatory pathways. For instance, CO may enhance the activation efficiency of sGC by increasing the activity of NOS or stabilizing the effect of NO, forming a positive feedback regulation. This phenomenon suggests that CO is not only an independent signaling molecule but may also act as an important auxiliary factor of the NO pathway in the fine regulation of the erectile process.^73^,^74^

Current research on the relationship between CO and ED remains relatively limited. Compared with the extensive studies on NO in the mechanism of penile erection, the role of CO as another endogenous gas signaling molecule has not been fully revealed. However, with the development of gasotransmitter biology in recent years, more and more evidence indicates that CO plays an important physiological role in regulating vascular tone and neural conduction and may play a key role in the regulation of male sexual function. A study has shown that in animal models of ED induced by metabolic diseases such as diabetes and hyperlipidemia, the expression level of HO-2 in penile corpus cavernosum tissue is significantly downregulated. HO-2 is a key rate-limiting enzyme that catalyzes the decomposition of heme to generate CO.^75^ The reduction in its expression directly leads to insufficient endogenous CO synthesis, thereby affecting the normal activation of downstream signaling pathways. The deficiency of this bioactive gas is closely related to the significant decline in penile erectile function, manifested as decreased relaxation ability of corpus cavernosum smooth muscle, reduced arterial blood flow perfusion, and shortened erectile duration, among other typical pathological features.^75^ Notably, intervention experiments have shown that the administration of exogenous CO donors, such as CO-releasing molecule (CORM)-3, can restore the concentration of CO in penile tissue to a certain extent and improve the relaxation response of smooth muscle. In addition, upregulating the expression of HO-2 through gene transfection technology can also effectively enhance local CO production, thereby partially reversing ED in models of diabetes or aging-related ED.^76^ These results suggest that enhancing the HO-2/CO pathway may be a potential strategy for treating specific types of ED, especially in patients with chronic diseases accompanied by oxidative stress or nerve damage^77^ (**Figure 6**).

**Figure 6:**
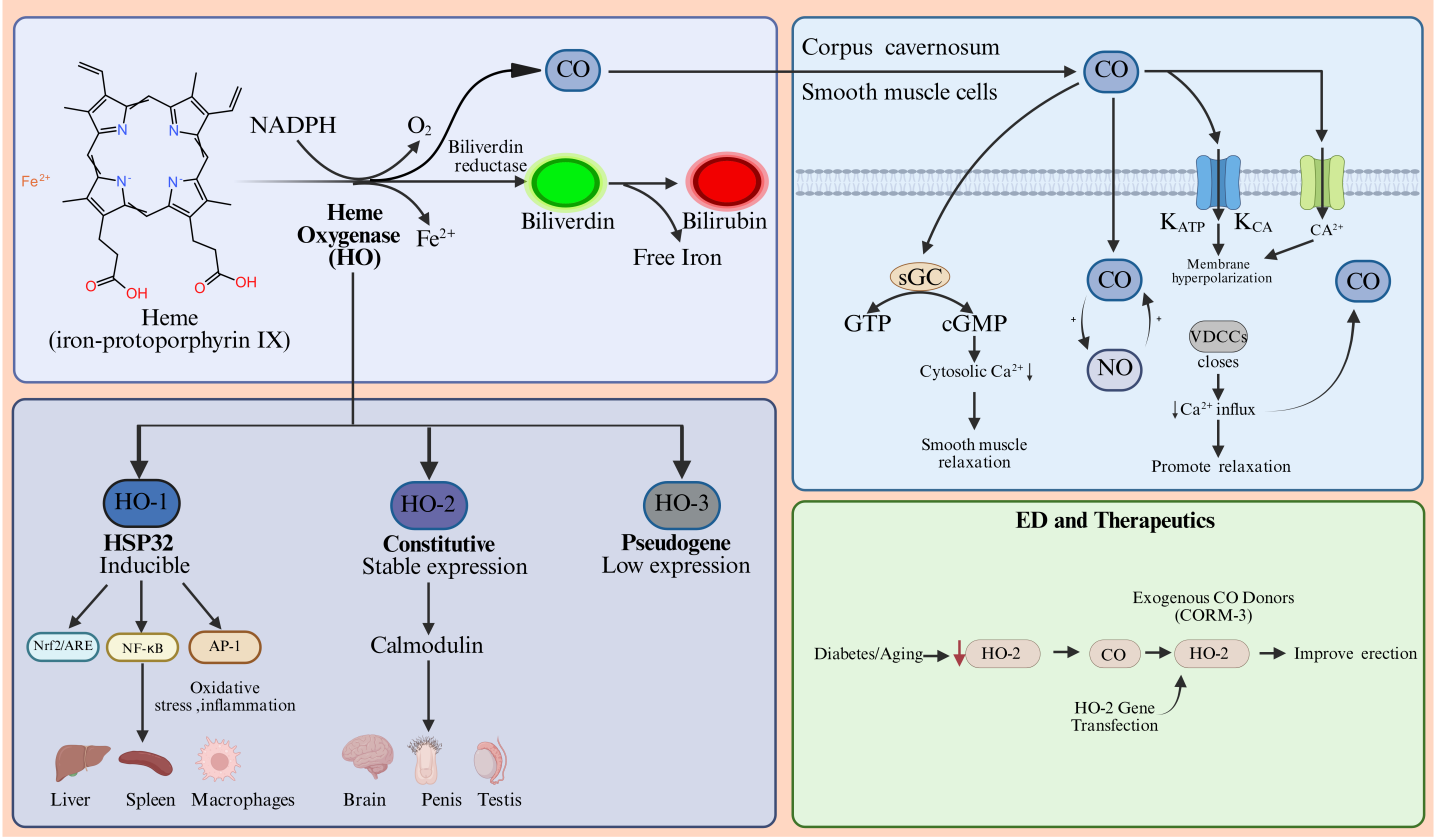
The core mechanism of CO in the corpus cavernosum of the penis. Lef: The process by which heme is converted into the bioactive molecule CO under the catalysis of different HO isoenzymes. Right: How CO induces membrane hyperpolarization by activating the sGC-cGMP pathway and opening potassium channels, a dual mechanism that lowers intracellular calcium concentration and thereby facilitates the molecular basis of corpus cavernosum smooth muscle relaxation. Created with BioRender.com. AP-1: Activator protein 1; ARE: antioxidant response element; cGMP: cyclic guanosine monophosphate; CO: carbon monoxide; CORM-3: carbon monoxide-releasing molecule 3; GTP: guanosine triphosphate; HO: heme oxygenase; HSP32: heat shock protein 32; K_ATP_: adenosine triphosphate-sensitive potassium channel; KCa: calcium-activated potassium channel; NADPH: nicotinamide adenine dinucleotide phosphate; NF-κB: nuclear factor kappa-B; NO: nitric oxide; Nrf2: nuclear factor erythroid 2-related factor 2; O_2_: oxygen; sGC: soluble guanylyl cyclase; VDCC: voltage-dependent calcium channel.

### Hydrogen sulfide

H_2_S is a gas molecule with a typical rotten egg smell and has long been regarded as a toxic gas. It is commonly found in industrial waste gas, decaying organic matter and intestinal fermentation processes.^78^ However, since the late 1990s, with the in-depth development of biomedical research, H_2_S has been gradually confirmed as the third endogenous gas signaling molecule following NO and CO, playing a significant role in regulating various physiological functions. This discovery has completely transformed the traditional perception of H_2_S, changing it from an “environmental toxin” to a “physiological regulatory factor,” and has attracted extensive attention in fields such as cardiovascular, nervous systems, inflammatory regulation, and energy metabolism.^79^,^80^

In the body, H_2_S is mainly produced through the metabolic reactions of sulfur-containing amino acids, especially L-cysteine and L-methionine, catalyzed by three key enzymes. These three enzymes are cystathionine β-synthase (CBS), cystathionine γ-lyase (CSE), and 3-mercaptopyruvate sulfurtransferase.^81^ CBS and CSE mainly rely on vitamin B6 as a cofactor and participate in the cascade reactions of the transsulfuration pathway. Among them, CBS is highly expressed mainly in brain tissue, liver and kidney, and plays an important role in maintaining neuronal excitability and antioxidant defense in the central nervous system; CSE is mainly distributed in vascular endothelial cells, smooth muscle cells and cardiac muscle tissue, and is closely related to vascular dilation and blood pressure regulation. In contrast, 3-mercaptopyruvate sulfurtransferase is expressed more widely, existing in the brain, kidneys, liver, pancreas, and even within mitochondria. Its catalytic process is usually coupled with α-ketoglutarate/glutamate metabolism, suggesting that it may play a unique role in mitochondrial energy metabolism and oxidative stress response.^82^

The generation of H_2_S is tissue-specific and dependent on physiological conditions. For instance, under hypoxic or inflammatory conditions, certain cells increase the production of H_2_S by up-regulating the expression of CSE or 3-mercaptopyruvate sulfurtransferase to exert its anti-inflammatory, antioxidant and cytoprotective effects.^83^,^84^,^85^ In addition, the gut microbiota has been confirmed to be capable of generating a certain amount of H_2_S through the metabolism of sulfur-containing proteins in the diet. Although this “exogenous” H_2_S has a relatively low concentration, it still plays a certain physiological role in maintaining the integrity of the intestinal barrier and regulating immune responses.^86^,^87^

At present, the metabolic pathways of H_2_S in the body have not been fully elucidated, but existing research indicates that its clearance mechanisms mainly include two major routes. The first is the oxidative metabolic pathway: H_2_S is gradually oxidized to sulfite (SO_3_^2−^) by sulfide oxidase in the mitochondria, and then further converted to sulfate (SO_4_^2−^), which is eventually excreted from the body through urine by the kidneys. This process not only detoxifies H_2_S but also participates in the recycling of sulfur elements.^88^,^89^ The second one is the combination and clearance pathway: H_2_S can combine with metal ions such as iron (Fe^2+^) and copper (Cu^2+^) to form insoluble sulfide precipitates, such as ferrous sulphide, thereby reducing its biological activity. In addition, some H_2_S can also combine with hemoglobin or cytochrome c oxidase to be temporarily stored or inactivated.^90^ Due to the dual biological characteristics of H_2_S, which exerts signaling and protective effects at low concentrations but shows cytotoxicity, such as inhibiting cytochrome c oxidase and disrupting the respiratory chain, at high concentrations, the body must maintain its concentration within the physiological range through a fine-tuned synthesis and clearance mechanism to achieve a dynamic balance. This “concentration window effect” makes H_2_S play a complex role in the occurrence and development of various diseases, potentially being a pathological factor or a therapeutic target.^91^

H_2_S plays a significant regulatory role in penile erection. As an endogenous gas signaling molecule identified after NO and CO, its importance in the physiological regulation of the urogenital system has been increasingly recognized in recent years. Research indicates that H_2_S not only participates in cardiovascular dilation, neural transmission, and anti-inflammatory responses but also exerts a crucial multi-target regulatory effect on male erectile function. It can coordinate the regulation of the tension state of the corpus cavernosum smooth muscle through multiple mechanisms, promoting blood inflow and retention, thereby supporting the initiation and maintenance of normal erectile processes.^92^,^93^

Specifically, H_2_S can directly act on the smooth muscle cells of the penile corpus cavernosum, inducing their relaxation, thereby promoting increased arterial blood flow and filling of the cavernous spaces, and achieving effective penile erection.^92^ This effect is not dependent on a single pathway but is achieved through multiple interrelated molecular mechanisms. Firstly, H_2_S can activate ATP-sensitive potassium (K_ATP_) channels, which are important ion channels widely present in the cell membranes of blood vessels and smooth muscle cells. When H_2_S binds to them, it can cause the cell membrane to hyperpolarize, increasing the negative value of the membrane potential, thereby reducing the open probability of voltage-dependent calcium channels, such as L-type calcium channels, and decreasing the influx of extracellular calcium. Since intracellular calcium ion concentration is a core factor in regulating smooth muscle contraction, its decrease directly impedes the sliding of myofilaments, ultimately leading to smooth muscle relaxation.^94^ This mechanism has been confirmed by multiple *in vitro* tissue experiments. In the corpus cavernosum strip models of rats and rabbits, exogenous H_2_S donors can induce dose-dependent relaxation responses, and this effect can be significantly inhibited by the K_ATP_ channel blocker glibenclamide. Secondly, H_2_S can also produce a synergistic effect with the classic NO-cGMP signaling pathway, forming a “gas signaling network.” NO, as the core medium for erectile regulation, promotes cGMP production by activating sGC, and then activates PKG, triggering downstream relaxation effects.^94^,^95^ Research has found that H_2_S can enhance the phosphorylation level of eNOS, thereby increasing its activity and promoting the synthesis of NO. Additionally, H_2_S may also prolong the half-life of cGMP by inhibiting the activity of PDE5 or stabilizing the redox state of sGC, thus extending the duration of its signaling. This “synergistic” effect on the NO pathway enables H_2_S to partially compensate for erectile function even in pathological conditions such as diabetes or aging, where NO production is impaired, demonstrating potential therapeutic value.^7^ In addition, H_2_S can also affect the calcium sensitivity regulation mechanism of smooth muscle by interfering with the RhoA/Rho kinase signaling pathway. Under resting conditions, the RhoA/Rho kinase pathway is in a low-activity state; however, in certain diseases such as hypertension, diabetes, or psychogenic ED, this pathway is abnormally activated, leading to the inhibition of myosin light chain phosphatase, which results in a stronger contraction response to the same concentration of calcium ions.^96^ H_2_S has been proven to inhibit the activation of RhoA and the expression of its downstream Rho kinase, relieve the inhibition of myosin light chain phosphatase, restore its dephosphorylation ability, thereby reducing the sensitivity of smooth muscle to calcium ions and promoting a more sustained relaxation state.^97^ This mechanism is particularly significant in maintaining the stability of an erection in the later stage and preventing premature flaccidity.

In addition to the direct vasodilatory effect mentioned above, H_2_S also has significant antioxidant and anti-inflammatory properties, which play a crucial role in protecting the structural integrity of penile tissue.^98^ In models of ED related to diabetes, hyperlipidemia or aging, long-term hyperglycemia, dyslipidemia or chronic inflammation can lead to excessive accumulation of ROS, causing oxidative stress, damaging endothelial cells and nerve endings, and disrupting the microenvironment of the corpus cavernosum.^93^ H_2_S can alleviate oxidative damage by eliminating harmful free radicals such as superoxide anion and hydroxyl radical, and up-regulating the expression of antioxidant enzymes such as superoxide dismutase and glutathione peroxidase.^99^ At the same time, it can also inhibit the activation of the nuclear factor κB pathway, reduce the release of pro-inflammatory factors such as tumor necrosis factor-α and interleukin-6, alleviate local inflammatory responses, and thereby delay the fibrosis of the corpus cavernosum and the degeneration of blood vessels.^100^ It is worth noting that the biosynthesis of H_2_S in penile tissue is mainly catalyzed by CSE, which is highly expressed in the corpus cavernosum of both humans and animals. Animal experiments have shown that CSE knockout mice have significantly reduced H_2_S levels, accompanied by weakened erectile responses, while administration of H_2_S donors, such as sodium hydrosulfide (NaHS) or GYY4137, can effectively improve ED in diabetic or castrated models. These results further support the functional role of the H_2_S-CSE axis in erectile physiology.^101^,^102^

In recent years, an increasing number of studies have demonstrated a close association between H_2_S and ED. In animal models of ED induced by common chronic diseases such as diabetes, hypertension, and atherosclerosis, researchers have generally observed a significant decline in the H_2_S biosynthesis capacity in the corpus cavernosum tissue. This reduction is not an accidental phenomenon but is closely related to the down-regulation of the expression levels of key synthetic enzymes, CSE and CBS.^100^ Among them, CSE is regarded as the main source of H_2_S production in penile tissue, highly expressed in the smooth muscle of the corpus cavernosum and vascular endothelial cells; while CBS is more distributed in neural tissues and may be involved in the regulation of neurogenic erection. In a diabetic rat model, hyperglycemia-induced oxidative stress can inhibit the promoter activity of CSE, leading to a decrease in enzyme expression and thereby weakening the generation of H_2_S.^100^ Similarly, in aging or castrated animal models, a decline in androgen levels has also been confirmed to affect the transcriptional regulation of CSE, further exacerbating H_2_S deficiency. Notably, intervention experiments have shown that by administering exogenous H_2_S donors, such as NaHS, or the sustained-release H_2_S-releasing molecule GYY4137, local H_2_S concentrations can be elevated in a short period of time, effectively restoring the relaxation function of the corpus cavernosum tissue. These compounds can mimic the effects of endogenous H_2_S, activating K_ATP_ channels, causing cell membrane hyperpolarization, reducing calcium ion influx, and synergistically enhancing the activity of the NO-cGMP signaling pathway, thereby improving penile arterial blood flow perfusion and corpus cavernosum filling.^102^,^103^ In addition, gene therapy strategies have also shown promise: introducing the CSE gene into the corpus cavernosum tissue via adeno-associated virus vectors can stably and long-term upregulate the expression of H_2_S synthase, significantly improving erectile responses in models of diabetes or aging-related ED, suggesting that targeted regulation of the H_2_S generation pathway holds great therapeutic potential.^104^ Mechanistically, H_2_S not only participates in the erectile process by directly relaxing smooth muscle, but also has multiple protective effects. It can eliminate excessive ROS, enhance the activity of antioxidant systems such as superoxide dismutase and glutathione, and reduce the damage of oxidative stress to the microvessels and nerve endings in the corpus cavernosum. Meanwhile, H_2_S can also inhibit the activation of the nuclear factor κB pathway, reduce the expression of pro-inflammatory factors such as tumor necrosis factor-α and interleukin-6, alleviate chronic inflammatory responses, and prevent corpus cavernosum fibrosis and structural remodeling. These effects collectively maintain the normal microenvironment of penile tissue, which is of great significance for the prevention of the occurrence and development of ED^100^,^105^,^106^ (**Figure 7**).

**Figure 7:**
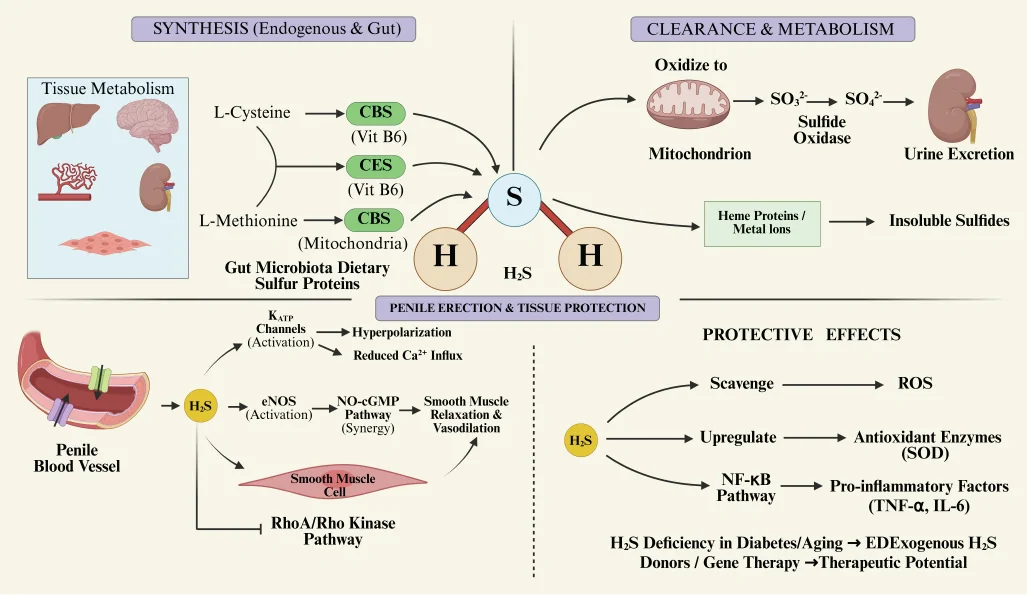
The metabolic pathways and mechanisms of H_2_S in penile erection and tissue protection. H_2_S is mainly endogenously produced in multiple tissues from L-cysteine and L-methionine under the enzymatic actions of CBS, CSE, and other enzymes, or generated through metabolism by the gut microbiota. Its clearance depends on mitochondrial oxidation to sulfate, which is then excreted in the urine. In penile tissue, H_2_S promotes smooth muscle relaxation and vasodilation by activating K_ATP_ channels to induce hyperpolarization, activating eNOS to produce a NO-cGMP synergistic effect, and inhibiting the RhoA/Rho kinase pathway. Additionally, H_2_S exhibits significant protective effects, including scavenging ROS, upregulating SOD, and inhibiting NF-κB-mediated inflammatory responses. For endogenous H_2_S deficiency caused by diabetes or aging, supplementation with donor drugs or gene therapy to restore H_2_S represents a potential strategy for treating ED. Created with BioRender.com. CBS: Cystathionine β-synthase; cGMP: cyclic guanosine monophosphate; CSE: cystathionine γ-lyase; ED: erectile dysfunction; eNOS: endothelial nitric oxide synthase; H_2_S: hydrogen sulfide; IL-6: interleukin-6; K_ATP_: adenosine triphosphate-sensitive potassium channel; NF-κB: nuclear factor kappa-B; NO: nitric oxide; ROS: reactive oxygen species; SOD: superoxide dismutase; TNF-α: tumor necrosis factor-alpha; Vit B6: vitamin B6.

## Research Progress of Gas Signal Molecules in Erectile Dysfunction Treatment

### Nitric oxide-based treatment strategies

NO donors are among the earliest drug categories applied in the treatment of ED. Their research can be traced back to the exploration of vasoactive substances in the mid-20^th^ century. With the confirmation of NO as an endothelium-derived relaxing factor in the 1980s and its establishment of a core position in the mechanism of penile erection, the scientific community began to attempt to restore impaired erectile function by supplementing NO exogenously. Traditional NO donors such as nitroglycerin and sodium nitroprusside were thus introduced into clinical research on ED.^107^,^108^ PDE5 is the main enzyme that degrades cGMP, and its activity can be regulated by cGMP through an allosteric mechanism. PDE5i, such as Sildenafil, delays the degradation of cGMP by competitively binding to the catalytic site of PDE5, thereby enhancing the smooth muscle relaxation effect mediated by NO. This is precisely the core mechanism for treating ED.^109^,^110^,^111^ These drugs can release NO in the body through non-enzymatic reactions or metabolic transformation, activating sGC in the smooth muscle cells of the penile corpus cavernosum, thereby promoting the synthesis of cGMP. As a key second messenger, cGMP can activate PKG, triggering downstream cascades, including reducing intracellular calcium ion concentration, opening potassium channels, and inhibiting the RhoA/Rho kinase signaling pathway. Ultimately, this leads to smooth muscle relaxation, arterial dilation, and corpus cavernosum engorgement, completing the process of penile erection.^112^

However, despite the clear pharmacological basis of traditional NO donors, their clinical application still faces multiple challenges. Firstly, drugs such as nitroglycerin have an extremely short half-life, with their effects typically lasting no more than 30 minutes, making it difficult to maintain a stable erectile state. Secondly, their administration routes mostly rely on intravenous injection or sublingual administration, with significant fluctuations in bioavailability and a high risk of causing systemic vasodilation.^113^,^114^ Because NO not only acts on the local penile vessels but also widely affects the coronary arteries, cerebral vessels and peripheral resistance vessel system, patients often experience adverse reactions such as headache, facial flushing, palpitations and even orthostatic hypotension. In patients with concomitant cardiovascular diseases or those using PDE5i, such as Sildenafil, severe hypotension may occur, increasing the risk of cardiovascular events. In addition, long-term use is prone to tolerance, and the dose needs to be gradually increased to maintain efficacy, further exacerbating safety concerns.^59^,^115^

In response to the above limitations, the development of new NO donors in recent years has become an important direction in the field of ED treatment, aiming to achieve more controllable, targeted and less side-effect NO delivery strategies. Among them, molsidomine, as an oral prodrug, shows good application prospects. The drug itself has no direct activity, but is rapidly metabolized in the liver into the active intermediate product SIN-1 (3-morpholinosydnonimine). SIN-1 can spontaneously decompose and continuously release NO under physiological conditions without the participation of reductase or thiol compounds, thereby avoiding the common drug resistance mechanism of traditional nitrate drugs.^116^ More importantly, SIN-1 releases NO at a relatively steady rate and its effect can last for several hours, which helps maintain a sustained erectile response and reduces the occurrence of significant hemodynamic fluctuations, significantly lowering the incidence of headache and hypotension.^117^ An animal experiment has confirmed that molsidomine can significantly increase the cGMP level in the penile tissue of diabetic rats and castrated model rabbits, improve the conduction velocity of the cavernous nerve and the ratio of smooth muscle to collagen, and enhance the erectile response induced by electrical stimulation.^118^ In clinical research, randomized double-blind controlled trials have shown that ED patients who took mosapride orally once a day for 4 to 8 weeks had significant improvements in the total score of the International Index of Erectile Function and the sub-scale score of sexual satisfaction during intercourse. Color Doppler ultrasound also found that the maximum systolic velocity of the penile artery increased significantly, suggesting that penile blood perfusion was effectively improved.^119^ Meanwhile, clinical data show that its overall adverse event rate is lower than that of traditional NO donors. Most patients only report mild dizziness or gastrointestinal discomfort, and the symptoms usually subside rapidly after discontinuation of the medication.^120^ It is worth noting that the design concept of new NO donors is constantly evolving. Besides molsidomine, researchers are developing complex formulations with tissue-targeting properties, such as hybrid molecules formed by covalently linking NO with PDE5i, or using nanocarriers to achieve sustained release and local delivery of NO in the corpus cavernosum, aiming to enhance efficacy while minimizing systemic exposure as much as possible.^121^ In addition, multifunctional donors that combine NO release and antioxidant functions, such as NO complexes combined with vitamin E derivatives, have also been proposed, aiming to address the oxidative stress microenvironment that is prevalent in ED, protect endothelial cell function, and delay the progression of the disease.^122^

Directly activating NOS to promote the synthesis and release of endogenous NO is also a potential strategy for treating ED. By enhancing NOS activity, the local NO concentration in the penile corpus cavernosum can be increased, which in turn activates the sGC-cGMP signaling pathway, induces smooth muscle relaxation and vascular dilation, and thereby improves erectile function.^123^ Currently, some NOS agonists have been studied in animal experiments, among which 5-aminoimidazole-4-carboxamide ribonucleotide (AICAR) is a representative compound. AICAR can increase the expression of eNOS and enhance its phosphorylation level by activating adenosine 5’-monophosphate-activated protein kinase, thereby promoting the activity of eNOS and increasing the production of NO in the corpus cavernosum tissue, improving erectile function. Studies have shown that in animal models of ED related to diabetes or aging, AICAR can significantly increase cGMP levels and penile blood flow, and restore the erectile response induced by electrical stimulation. However, due to the wide participation of adenosine 5′-monophosphate-activated protein kinase in various physiological processes, such as energy metabolism, cell proliferation and inflammatory regulation, the mechanism of action of AICAR is systemic and multi-effect, which may lead to non-target effects; in addition, long-term use may cause potential adverse reactions such as hypoglycemia, elevated liver enzymes or abnormal immune regulation. Therefore, although this type of drug shows certain therapeutic potential, more in-depth basic and preclinical research is still needed in terms of specificity, safety and dosing regimens.^124^,^125^

### Carbon monoxide-based treatment strategies

CO donors are key tools for studying the application of the endogenous gas signaling molecule CO in the treatment of ED. For a long time, CO has been regarded as a toxic gas, mainly by binding with hemoglobin to form carboxyhemoglobin, reducing the oxygen-carrying capacity of blood and causing tissue hypoxia.^126^ However, since the 1990s, with the in-depth exploration of the biological functions of gas signal molecules, it has gradually been recognized that CO is not only the main product of heme degradation catalyzed by HO-1, but also an important endogenous messenger molecule that plays a key role in various physiological processes, such as vasodilation, anti-inflammation, anti-apoptosis and cell protection. In recent years, its potential value in the male reproductive system, especially in the regulation of penile erection, has also attracted widespread attention.^127^ At present, a variety of compounds capable of regulating the release of CO have been developed, collectively known as CORMs. Among them, CORM-2 and CORM-3 are the most representative first-generation metal-based CO donors. CORM-2 is a ruthenium carbonyl complex with relatively low water solubility, which can slowly release CO through a non-enzymatic mechanism under physiological conditions. In contrast, CORM-3 has higher water solubility and faster release kinetics, making it suitable for intravenous injection or local perfusion administration. Additionally, second-generation CORMs, such as CORM-A1 (borane-based), do not contain transition metals and rely on pH or specific enzyme environments to control CO release, offering better biocompatibility and tissue selectivity. The design goal of these CO donors is to deliver low concentrations of CO in a controlled and sustained manner without causing systemic toxicity, thereby mimicking the physiological effects of endogenous CO.^128^,^129^

Animal experiments have shown that administration of CO donors can significantly improve erectile function in animal models of ED under pathological conditions such as diabetes, hyperlipidemia, aging and castration. For instance, in a streptozotocin-induced diabetic rat model, intravenous injection of CORM-3 significantly increased intracavernous pressure in a dose-dependent manner.^130^ Similar results were also verified in high-fat diet-fed mice and elderly rabbit models. These functional improvements are not only manifested as an increase in the amplitude of erectile responses, but also include the coordinated improvement of structural and functional indicators such as accelerated nerve conduction velocity, restored endothelial integrity, and optimized smooth muscle/collagen fiber ratio.^131^ The mechanism of action may involve multiple levels: on the one hand, CO can directly activate sGC in the smooth muscle cells of the penile corpus cavernosum, although its affinity for sGC is lower than that of NO. However, in the absence of NO or when sGC is inactivated due to oxidation, CO can still partially maintain the production of cGMP, thereby activating PKG, triggering downstream cascades, and ultimately inducing smooth muscle relaxation and arterial dilation. On the other hand, CO can also regulate the open state of potassium ion channels, such as KATP channels, causing cell membrane hyperpolarization, inhibiting the activity of voltage-gated calcium channels, and reducing calcium ion influx, thereby synergistically enhancing the dilatory effect. Notably, CO and NO have functional complementarity in the signaling pathway, especially in pathological conditions such as diabetes or atherosclerosis with endothelial dysfunction. When the NO-cGMP pathway is impaired, the CO-sGC-cGMP pathway may act as a compensatory mechanism.^74^ In addition, CO has significant anti-inflammatory and antioxidant properties, which endow it with tissue-protective potential in the chronic pathological process of ED. Chronic inflammation and oxidative stress are the common pathogenic bases of various etiological types of ED, which can lead to endothelial cell damage, neurodegeneration and corpus cavernosum fibrosis.^132^ Studies have shown that CO can inhibit the activation of the nuclear factor κB pathway, down-regulate the expression of pro-inflammatory factors such as tumor necrosis factor-α and interleukin-1β, and at the same time, up-regulate the expression levels of antioxidant enzymes such as superoxide dismutase and HO-1, effectively eliminating excessive ROS, reducing lipid peroxidation and mitochondrial dysfunction.^133^ These effects help maintain the homeostasis of the penile microenvironment and delay the occurrence and development of structural lesions.

### Hydrogen sulfide-based therapeutic strategies

H_2_S donors are currently the main approach for studying the application of H_2_S in the treatment of ED. Common H_2_S donors, such as NaHS and GYY4137, have been widely used in various animal models of ED.^103^ Studies have shown that exogenous administration of H_2_S donors can significantly improve erectile function in animal models of ED induced by chronic diseases such as diabetes and hypertension. The mechanism of action involves multiple key signaling pathways: on the one hand, H_2_S can cause hyperpolarization of the smooth muscle cell membrane in the corpus cavernosum by activating KATP channels, reducing calcium ion influx, and thereby promoting smooth muscle relaxation; on the other hand, H_2_S upregulates eNOS activity, enhance the function of the NO-cGMP signaling pathway, and inhibit the excessive activation of the RhoA/Rho kinase signaling pathway, alleviating vascular contraction and fibrosis.^99^,^134^ These multi-target effects collectively promote the dilation of the corpus cavernosum and the recovery of blood flow perfusion. Clinical research has also preliminarily confirmed the safety and potential efficacy of H_2_S donors in the treatment of ED. For instance, a small-sample randomized controlled trial demonstrated that local application of H_2_S donors could significantly increase the International Index of Erectile Function scores of patients, improve parameters of nocturnal penile tumescence monitoring, and no obvious adverse reactions or abnormal laboratory indicators were observed throughout the treatment period.^135^ However, the current H_2_S donors still have problems such as short half-life, uncontrollable release kinetics, and poor tissue targeting. Their stability and pharmacokinetic properties need to be further optimized to enhance bioavailability and clinical translation potential.

Regulating the activity or expression level of H_2_S synthase can effectively control the production of endogenous H_2_S, and thus serves as a potential strategy for treating ED. H_2_S is mainly produced by three enzymes, among which CBS and CSE are highly expressed in the corpus cavernosum tissue and participate in maintaining normal erectile function. Studies have shown that up-regulating the expression of CBS or CSE through gene therapy or drug intervention can significantly increase the local H_2_S concentration and improve erectile function under pathological conditions such as diabetes and aging.^136^ For instance, in animal models of diabetes-induced ED, transfection of the CBS-encoding gene into the corpus cavernosum tissue via viral vectors can effectively increase the expression level of CBS protein and the production of H_2_S, accompanied by a significant increase in intracavernous pressure, suggesting the restoration of erectile function. These effects not only contribute to smooth muscle relaxation and vasodilation but may also exert tissue-protective actions through anti-inflammatory and antioxidant mechanisms, demonstrating potential therapeutic value for ED. However, due to the widespread expression of CBS and CSE in various tissues, their systemic upregulation may trigger non-target organ effects; moreover, H_2_S is cytotoxic at high concentrations and may inhibit mitochondrial respiratory chain function. Therefore, the mechanism of action of H_2_S synthase modulators is rather complex, and further in-depth basic and clinical research is needed in terms of specificity, dose control, and long-term safety.^137^,^138^

The research on gas signal molecules in the field of ED treatment has undergone decades of development and achieved several milestone breakthroughs. To clearly compare the characteristics and therapeutic potential of the three gas signal molecules, this article summarizes their core features as shown in **Table 1**.^56^,^61^,^91^,^103^,^139^,^140^,^141^,^142^,^143^,^144^,^145^,^146^

**Table 1:** Characteristics and potential of three gas signal molecules in ED treatment

	NO	CO	H_2_S
Endogenous synthetic enzymes	NOS (nNOS, eNOS, and iNOS)^139^	HO (HO-1, and HO-2)	CSE, CBS, and 3-MST^140^
Core mechanism	Activate sGC; increase cGMP^56^	Activate sGC; open K^+^ channels^141^	Open K_ATP_ channels; enhance the NO pathway; inhibit RhoA/ROCK^142^
Main physiological functions	Vasodilation and neurotransmission^143^	Vasodilation, anti-inflammation, anti-apoptosis	Vasodilation, antioxidation, anti-inflammation, cell protection
Therapeutic advantages	A core molecule for erection^144^	Partially activate sGC under oxidative stress, strong tissue protective effect^145^	Multi-target, strong antioxidant stress capacity, potential for neurogenic ED^103^
Main challenges	Extremely short half-life, easily quenched by ROS, systemic side effects	Narrow therapeutic window, potential toxicity concerns, difficult delivery control^61^	Concentration dual effect, release kinetics control, poor targeting^91^
Most promising directions	Targeted/sustained-release NO donors^146^	New safe CORMs; for refractory ED in diabetes, etc.	slow-release H_2_S donor; combination with stem cell therapy; nerve repair

3-MST: 3-Mercaptopyruvate sulfurtransferase; CBS: cystathionine β-synthase; cGMP: cyclic guanosine monophosphate; CO: carbon monoxide; CORM: carbon monoxide-releasing molecule; CSE: cystathionine γ-lyase; ED: erectile dysfunction; eNOS: endothelial nitric oxide synthase; H_2_S: hydrogen sulfide; HO: heme oxygenase; iNOS: inducible nitric oxide synthase; K_ATP_: adenosine triphosphate-sensitive potassium channel; nNOS: neuronal nitric oxide synthase; NO: nitric oxide; NOS: nitric oxide synthase; ROCK: Rho-associated protein kinase; ROS: reactive oxygen species; sGC: soluble guanylyl cyclase.

## Limitations and Future Prospects

Although gas signaling molecules represented by NO, CO and H_2_S have opened up highly promising new avenues for the treatment of ED, and significant progress has been made in related basic research and clinical exploration, there are still many challenges in translating them into safe and effective clinical therapies. Future research should focus on solving several key scientific problems and further clarifying the development direction.

### Main challenges

#### The complexity and interactions of molecular mechanisms

Gas signaling molecules do not act in isolation during the physiological and pathological processes of erection, but rather form a sophisticated regulatory network. For instance, H_2_S can enhance the biological effects of NO, while CO participates in regulating the balance between the HO/CO system and the NOS/NO system. The specific patterns of these networked interactions in ED caused by different types of etiologies, such as diabetes, nerve injury, and aging, have not been fully elucidated.^147^ Additionally, the biological effects of gas molecules exhibit a dual concentration-dependent characteristic: at low concentrations, they may exert cytoprotective effects, but at high concentrations, they may induce growth inhibition or cytotoxicity, which poses significant challenges in determining their therapeutic window.^148^

#### Optimization of pharmacokinetics and safety of donor compounds

At present, most gas molecule donors, such as CORM-2 and NaHS, are still in the preclinical research stage. Their metabolic pathways, bioavailability, tissue distribution, and potential long-term toxicity in the body have not been fully evaluated. For instance, some CORMs may release toxic by-products, and the release rate of some H_2_S donors is difficult to effectively control. Therefore, designing and synthesizing new, highly stable, and low-toxicity gas donors, and systematically evaluating their pharmacokinetic and pharmacodynamic characteristics are necessary steps to advance them towards clinical trials.

### Future directions and prospects

#### Development of novel intelligent delivery systems

Gas signaling molecules, characterized by their low molecular weight, rapid diffusion, and extremely short half-life, present significant challenges for achieving precise and controllable release *in vivo*. Conventional systemic administration, such as oral or intravenous delivery of gas donors, often fails to maintain effective therapeutic concentrations within the penile corpus cavernosum and is frequently limited by systemic side effects, including hypotension and headaches. To overcome these barriers, future research should prioritize the development of intelligent delivery platforms, such as nanoparticles, hydrogels, or microspheres, to enhance the efficacy of targeted therapies. By engineering these carriers to respond to specific pathological microenvironments and incorporating active targeting of penile endothelial or smooth muscle cells, “on-demand” release can be achieved. Integrating these advanced systems with local or transdermal administration routes represents a pivotal strategy to maximize local therapeutic efficacy while minimizing systemic exposure and enhancing overall safety.

#### Exploration of multi-target combined treatment strategies

Given the intrinsic interconnectivity of the gas signaling molecule network and its complementary role to existing therapies, combined treatment strategies show great promise. Specifically, this includes the combined application of different gas donors, such as H_2_S donors and NO donors, to achieve a synergistic effect; the combination of gas donors with PDE5i to enhance cGMP levels through a dual mechanism of increasing production and reducing degradation, offering new treatment options for patients who are ineffective or intolerant to PDE5i; and the integration of gas molecule therapy with emerging technologies such as stem cell therapy, low-intensity extracorporeal shock wave therapy, or gene therapy, adopting a multi-target intervention strategy to systematically restore impaired erectile function.

#### In-depth exploration of the value of gas molecules in refractory ED

For refractory ED with limited current treatment options, such as diabetic ED, ED after radical prostatectomy, and ED after pelvic radiotherapy, gas signaling molecules may exhibit unique therapeutic advantages due to their multiple biological effects, such as vasodilation, anti-inflammation, antioxidation, anti-apoptosis, and promotion of nerve regeneration. Preclinical mechanism studies should be conducted in depth for these specific patient populations, and strict randomized controlled clinical trials should be implemented to confirm their efficacy and safety.

#### Strengthening clinical translation and accumulation of evidence-based medical evidence

The ultimate goal is to promote promising gas donors from laboratory research to clinical application. This requires close collaboration among industry, academia, research institutions, and medical professionals to systematically conduct preclinical safety evaluations and Phase I to III clinical trials in accordance with established norms. Through large-scale, multi-center, and long-term follow-up clinical studies, high-quality evidence-based medical evidence should be accumulated to clarify the efficacy, safety, and optimal application schemes of various gas molecule therapies in different ED patient populations. In summary, although gas signal molecule therapy has broad prospects, it still faces multiple challenges in moving towards clinical application.

## Conclusion

Although gas signaling molecules show great potential in the treatment of ED, their clinical translation is still limited by delivery bottlenecks caused by short molecular half-life and high diffusivity, as well as the strict requirements for dose-effect control and the heterogeneity of ED pathology. Current experimental designs also have limitations, mainly manifesting as an overreliance on animal models while neglecting interspecies anatomical differences, and a lack of long-term in vivo safety evaluation and real-time monitoring of local concentrations. Therefore, it is urgent to achieve precise targeting and “on-demand release” through multidisciplinary approaches using responsive nanocarriers (such as nanoparticles and hydrogels), and to develop novel stable gas donors. Combining omics technologies to carry out personalized precision medicine will facilitate the transition of this therapy from basic research to clinical application, offering new treatment hope for patients with refractory ED.

Gas signal molecule therapy represents a dynamic frontier in the field of ED treatment. In recent years, with the in-depth study of the biological functions of endogenous gas molecules, such as NO, H_2_S, and CO, it has been gradually recognized that these small molecules not only play a key role in intercellular communication but also have significant value in regulating penile corpus cavernosum smooth muscle relaxation, vasodilation, anti-inflammation, anti-oxidative stress, and neuroprotection. Although traditional treatment methods such as PDE5i are effective for the majority of patients, their efficacy is limited in patients with refractory ED, such as those with diabetes, post-prostate surgery, or nerve injury. Gas signal molecules, with their multi-target and multi-pathway mechanisms, show potential therapeutic advantages.

**Facts**

1. Phosphodiesterase type 5 inhibitors fail in 30–40%, especially in refractory erectile dysfunction (ED) where the nitric oxide (NO) pathway is severely damaged.
2. Research now targets a multi-gas network involving carbon monoxide (CO) and hydrogen sulfide (H_2_S), which are also endogenous vasodilators.
3. CO and H_2_S can bypass/synergize with NO; e.g., H_2_S activates adenosine triphosphate-sensitive potassium channels to induce relaxation.
4. Nanocarriers and prodrugs enable targeted, “on-demand” release to overcome short half-life & toxicity.
5. Boosting these gas pathways reverses ED in animal models of diabetes, hyperlipidemia & nerve damage.

**Open Questions**

1. How do NO, CO and H_2_S interact as a network during different ED stages?
2. What are the precise therapeutic windows given their dual protective/cytotoxic effects?
3. What is the long-term metabolism, bioavailability & chronic toxicity of gas donors in humans?
4. Can we find biomarkers to identify ED subtypes best suited for gas-based *vs*. traditional therapy?
5. How to refine nanocarriers for truly intelligent, pathology-triggered release without systemic side-effects?

## Data Availability

*Not applicable.*
